# The impact of gut microbiota metabolites on cellular bioenergetics and cardiometabolic health

**DOI:** 10.1186/s12986-021-00598-5

**Published:** 2021-07-15

**Authors:** Lenka Tomasova, Marian Grman, Karol Ondrias, Marcin Ufnal

**Affiliations:** 1 Institute of Clinical and Translational Research, Biomedical Research Center, Slovak Academy of Sciences, Dubravska cesta 9, 845 05 Bratislava, Slovak Republic; 2 Department of Experimental Physiology and Pathophysiology, Laboratory of Centre for Preclinical Research, Medical University of Warsaw, 02-091 Warsaw, Poland

**Keywords:** H_2_S, SCFAs, Nitrate, Nitric oxide, TMAO, Mitochondria, Metabolic syndrome

## Abstract

Recent research demonstrates a reciprocal relationship between gut microbiota-derived metabolites and the host in controlling the energy homeostasis in mammals. On the one hand, to thrive, gut bacteria exploit nutrients digested by the host. On the other hand, the host utilizes numerous products of gut bacteria metabolism as a substrate for ATP production in the colon. Finally, bacterial metabolites seep from the gut into the bloodstream and interfere with the host’s cellular bioenergetics machinery. Notably, there is an association between alterations in microbiota composition and the development of metabolic diseases and their cardiovascular complications. Some metabolites, like short-chain fatty acids and trimethylamine, are considered markers of cardiometabolic health. Others, like hydrogen sulfide and nitrite, demonstrate antihypertensive properties. Scientific databases were searched for pre-clinical and clinical studies to summarize current knowledge on the role of gut microbiota metabolites in the regulation of mammalian bioenergetics and discuss their potential involvement in the development of cardiometabolic disorders. Overall, the available data demonstrates that gut bacteria products affect physiological and pathological processes controlling energy and vascular homeostasis. Thus, the modulation of microbiota-derived metabolites may represent a new approach for treating obesity, hypertension and type 2 diabetes.

## Introduction

Metabolic diseases and their cardiovascular complications are the primary cause of morbidity and mortality in affluent societies. The last few decades have seen a fundamental transformation of dietary patterns. There is a notable increase in the consumption of high saturated fat, simple carbohydrates and salt. A decrease in physical activity accompanies these dietary changes. The two together disrupt the energy homeostasis in humans, resulting in a dramatic increase in the prevalence of metabolic and cardiovascular disorders such as obesity, diabetes and hypertension [1]. Interestingly, recent studies suggest that alterations in gut microbiota composition and increased gut permeability accompany these lifestyle-associated disorders [2–4]. These changes lead to excessive leakage of gut metabolites into the circulation [5, 6].

**Fig. 1:**
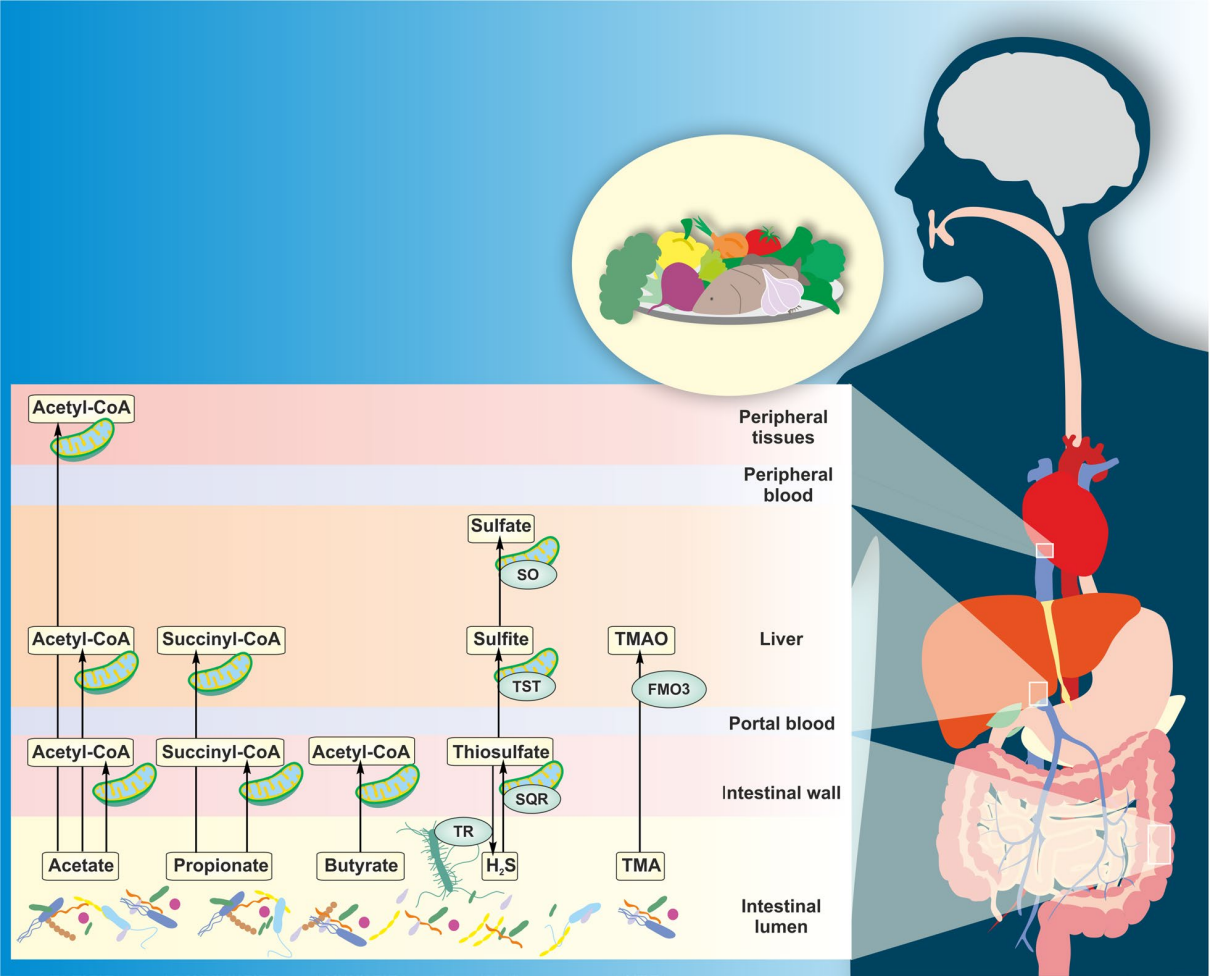
Metabolism of gut microbiota products from dietary substrates. Dietary subtrates enter the gastrointestinal tract and are further processed by gut microbes to various metabolites, including SCFAs (acetate, propionate, butyrate), H_2_S and TMA. The majority of butyrate is oxidized to acetyl-CoA in the mitochondria of colonic epithelial cells. Propionate and acetate are partially metabolized in the gut. Though, the majority of propionate is oxidized to succinyl-CoA in the liver. From SCFAs, acetate reaches the highest concentrations in peripheral blood, thus contributing mostly to the systemic production of energy in the peripheral tissues. Additionally, colonic mucosa oxidizes H_2_S by the action of mitochondrial SQR to thiosulfate. Thiosulfate may be either recycled to H_2_S by the action of microbial TR in the lumen of the gut or reach liver for further oxidation to sulfite and sulfate by mitochondrial TST and SO respectively. Likewise, the fraction of TMA that reaches the liver is oxidized to TMAO by cytosolic FMO3. CoA; *coenzyme A*, SQR; *sulfide quinone reductase*, TR; *thiosulfate reductase*, TST; *thiosulfate sulfurtransferase,* SO*; sulfite oxidase*, TMA; *trimethylamine*, TMAO; *trimethylamine N-oxide,* FMO3; *flavin-containing monooxygenase 3*

The mammalian gut is colonized by a complex microbial community composed of bacteria, archaea, fungi, helminths and protozoa. Most abundant are bacteria (~ 10^12^ cells/g of stool), followed by archaea (~ 10^9^ cells/g of stool) and fungi (~ 10^4^ cells/g of stool) [7, 8]. The composition of gut microbes is shaped more by environmental factors, such as mode of delivery, diet and geography than genetics [9]. The relationship between microbiota and host is commensal under physiological conditions. On the one hand, the host provides habitat and nutrition for the growth of gut microbes. On the other, commensal microbes regulate the gut's homeostasis, metabolize nutrients, bile acids, xenobiotics, and toxins [10]. Importantly, microbiota represents the largest endocrine organ in the human body by producing various biologically active compounds (Fig. 1). Gut-derived metabolites may act locally in the gut or signal distant organs via systemic circulation.

Accumulating evidence points to the prominent role of gut microbiota products in the regulation of energy metabolism [11, 12]. Gut metabolites use both passive and active transport to reach the intracellular space. Once inside the cell, they can interfere with mitochondrial respiration, thereby controlling ATP production [11, 13–15]. In addition, gut products may alter the activity of membrane channels and receptors involved in regulating blood pressure, glucose and lipid metabolism, thus playing a key role in developing cardiometabolic disorders [16–18]. This review summarizes recently published studies investigating the influence of gut microbiota-derived metabolites on the regulation of cellular energy homeostasis and the development of cardiometabolic disorders.

## Microbiota metabolites as energy substrates and biological mediators

The colonization of the human gut by microbes results in creating a complex ecosystem unique to every individual. This living coating of the intestines is referred to as biofilm. Polymicrobial biofilms are formed at the surface of the gut epithelium or in the gut lumen attached to the mucus or food particles [19]. In comparison to tissue-forming cells, members of polymicrobial community have distinctive structures and metabolism, requiring a higher level of organization. The metabolic rate of gut bacteria depends on several factors, but mainly on substrate availability, intestinal transit time and redox balance [20, 21]. Therefore, diet plays an essential role in the formation and modulation of gut biofilms. Firstly, the composition of the microbial communities depends on the interactions occurring between members of the intestinal flora. For instance, some bacterial strains compete for the same substrate, making co-colonization difficult. Thus, co-colonization occurs between those species which produce substrates other strains can utilize. Secondly, polymicrobial biofilms are in direct contact with mucosal tissues of the colon. Hence, gut microbes and their products affect the metabolism of the colonic mucosa and vice versa. The mutual interactions between polymicrobial phenotypes and whole-body homeostasis have been intensively studied [19]. In the following paragraphs, we present several metabolites involved in the regulation of host energy metabolism. We provide physiological ranges of these metabolites in the intestines, their absorption rates and major commensal microbial producers (Table 1). We also discuss how they affect mitochondrial bioenergetics. For a detailed description of the effects of these metabolites on membrane channels and receptors involved in the regulation of energy and vascular homeostasis, please refer to previous reviews [17, 22–26].

**Table 1:** Substrates consumed by major commensal microbial species to produce metabolites in the human gut

Substrate	Genus (species)	Metabolite	References
Acetate	*Faecalibacterium (F. prausnitzii)* *Eubacterium (E. rectale, E. halii)* *Roseburia (R. intestinalis, R. hominis, R. inulinivorans)* *Anaerostipes (A. hadrus)* *Coprococcus (C. catus)*	Butyrate	[36–38]
Succinate	*Bacteroides (B. uniformis, B. vulgates)* *Prevotella (P. copri)* *Alistipes (A. putredinis)* *Ruminococcus (R. flavefaciens)* *Phascolarctobacterium (P. succinatutens)* *Dialister (D. invisus)* *Akkermansia (A. muciniphila)*	Propionate	[38]
Acrylate	*Megasphaera (M. elsdenii)* *Coprococcus (C. catus)*	Propionate	[38]
Fucose or rhamnose	*Roseburia (R. inulinivorans)* *Eubacterium (E. hallii)* *Blautia (B. obeum)*	Propionate	[38]
Pyruvate	*Bacteroides (B. frragilis, B. ovatus)* *Bifidobacterium (B. adolescentis, B. longum)* *Clostridium (C. perfringens, C. bifermentans)* *Ruminococcus (R. bromii, R. gnavus)*	Acetate	[42, 43]
Sulfate	*Desulfovibrio (D. piger, D. desulfuricans)*	H_2_S	[46]
Sulfite	*Escherichia (E. coli)*	H_2_S	[49]
Cysteine	*Escherichia (E. coli)* *Enterobacter (E. aerogenes, E. cloacae)*	H_2_S	[50]
Thiosulfate	*Citrobacter (C. freundii)* *Proteus (P. vulgaris)* *Edwardsiella (E. tarda)*	H_2_S	[56]
Nitrite	*Lactobacillus (L. acidophilus, L. shirota, L. rhamnosus)* *Bifidobacterium (B. bifidus, B. breve, B. infantis)*	NO	[81]
Arginine	*Bacillus (B. subtilis*, *B. anthracis*) *Deinococcus (D. radiodurans*)	NO	[82–84]
Formate	*Methaninobrevibacter (M. smithii)*	Methane	[95]
Methanol	*Methanosphaera (M. stadtmanae)*	Methane	[94]
Choline	*Anaerococcus (A. hydrogenalis)* *Clostridium (C. asparagiforme, C. hathewayi, C. sporogenes)* *Desulfitobacterium (D. hafniense)* *Escherichia (E. fergusonii)* *Proteus (P. penneri)* *Providencia (P. rettgeri, P. alcalifaciens, P. rustigianii)* *Edwardsiella (E. tarda)* *Yokenella (Y. regensburgei)*	TMA	[98, 99]
Carnitine	*Citrobacter (C. freundii)* *Escherichia (E. coli)* *Proteus (P. vulgaris)*	TMA	[99]

### Short-chain fatty acids

Dietary fermentation in the mammalian gut produces SCFAs. Since the p*K*_a_ of the SCFAs is ~ 4.8, at the colonic pH ~ 6, more than 90% is in its un-protonated form [27]. These small molecules may contain up to seven carbon atoms, straight or branched chain and include formate, acetate, propionate, butyrate, isobutyrate, valerate, isovalerate, 2-methylbutyrate, hexanoate and heptanoate. The substrates for the production of straight-chain fatty acids originate primarily from plants and include cellulose, pectins and oligosaccharides. The branched-chain fatty acids are formed by the degradation of amino acids valine, leucine and isoleucine. Fermentation of pectins and xylans produces acetate, while that of starch produces butyrate [28, 29]. The metabolism of arabinoglycans results in the production of acetate and propionate. The concentrations of SCFAs are highest in the colon and determined by substrate availability and microbiota composition in different segments, reaching 20–70 mmol/l in the distal colon and 70–140 mmol/l in the proximal colon [30]. With the increase in SCFAs concentration, pH value is decreasing due to the accumulation of H^+^. Importantly, pH value regulates microbiota composition and reduce the colonization by pathogenic species [31]. For instance, at pH 5.5, butyrate producers of Firmicutes phylum are dominant. In contrast, acetate and propionate producers of Bacteroidetes dominate at higher pH values over butyrate producers [32].

Furthermore, the availability of substrates regulates the fermentation process. For instance, *Bacteroides ovatus* accumulates succinate under high carbohydrate levels. However, it utilizes succinate for energy production and accumulates propionate under low carbohydrate levels. Similarly, *Clostridium perfringens* and *Bifidobacterium breve* generate lactate as an electron sink under excess carbohydrates and acetate or formate as products of energy metabolism under carbohydrate-limiting conditions [33]. The levels of SCFAs decrease from millimolar ranges in the colon to nanomolar ranges in the portal and peripheral blood. In healthy humans, the acetate, propionate and butyrate ratio is ~ 60/20/20 in the colon, ~ 69/23/8 in the portal blood and ~ 89/6/5 in the peripheral blood [27]. Diet composition affects the ratios of different SCFAs. For instance, a diet rich in carbohydrates and sugars increases the propionate/acetate ratio, whereas a high-fiber diet increases acetate levels [34, 35].

#### Butyrate

**Fig. 2:**
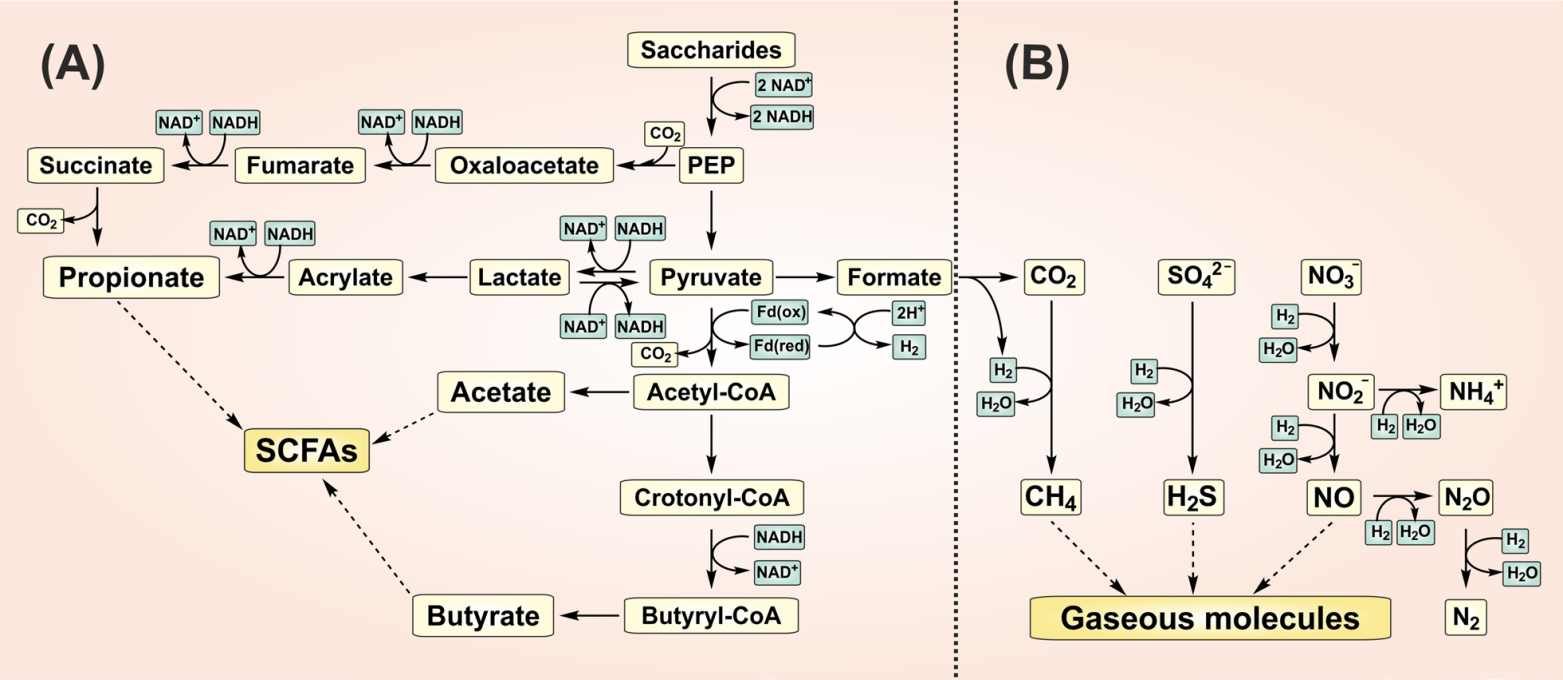
Schematic diagram of SCFAs and gaseous molecules production by gut microbes. **A** The fermentation of saccharides to PEP by gut microbes is coupled with the reduction of NAD^+^ to NADH. The metabolism of PEP to SCFAs (acetate, propionate and butyrate) is coupled with the regeneration of NAD^+^ and H_2_ production by Fd **B** Hydrogenotrophs utilize H_2_ to produce gaseous signaling molecules (H_2_S, CH_4_ and NO). Pathways have been simplified to highlight key end-products. PEP; *phosphoenolpyruvate*, Fd; *ferredoxin oxidoreductase*

Various gut bacteria, particularly the gram-positive anaerobic bacteria, *Faecalibacterium*, *Eubacterium* and *Roseburia* species from *Clostridial* clusters of Firmicutes generate butyrate [36–38]. These bacteria condense two molecules of acetyl-CoA to acetoacetyl-CoA, which subsequently converts to β-hydroxybutyryl-CoA and crotonyl-CoA. The final product is butyryl-CoA (Fig. 2A). This process consumes NADH. Butyryl-CoA may be further converted to butyrate by a butyryl-CoA:acetate Co-A transferase or phosphorylated to butyryl-phosphate and converted to butyrate by a butyrate kinase. Colon-derived SCFAs represent ~ 10% of the daily human caloric requirement [27]. SCFAs provide ~ 60–75% of energy for colonic epithelial cells, with butyrate as the primary contributor, followed by propionate and acetate [39]. Butyrate is converted in the mitochondria to butyryl-CoA by a butyryl-CoA synthetase. This process consumes ATP. Butyryl-CoA is subsequently transformed to crotonyl-CoA by a butyryl-CoA dehydrogenase. Crotonase catalyzes the formation of hydroxybutyryl-CoA, which then forms acetoacetyl-CoA, producing NADH, and then acetyl-CoA by an acetoacetyl-CoA thiolase. Colonic epithelial cells oxidize most of the colon-derived butyrate. The remaining butyrate is absorbed through the gut-blood barrier into the portal vein and metabolized by the liver. The absorption occurs from all colon segments at a rate of ~ 6–12 µmol/cm^2^/h, primarily by monocarboxylate transporters (MCT) [40]. These transporters exchange SCFAs for bicarbonates. This process neutralizes protons formed during SCFAs production and plays an essential role in regulating luminal pH. A minor portion of SCFAs is co-transported with H^+^ and Na^+^ cations. Only a fraction of the colon-derived butyrate reaches the peripheral circulation.

#### Propionate

Propionate may be produced in the gut in the process of carbohydrate fermentation either by succinate or acrylate pathway (Fig. 2A). The conversion of succinate to methylmalonate and further release of CO_2_ results in propionate production and is present in e.g., *Bacteroides, Prevotella, Alistipes, Ruminococcus*, *Phascolarctobacterium, Dialister, Akkermansia* species [38]. The conversion of pyruvate to lactate and acrylate is associated with the consumption of NADH. Acrylate may be further metabolized to propionate by the utilization of another NADH. The acrylate pathway is active in *Megasphaera* or *Coprococcus species* [38]. Propionate may also be derived from deoxy-sugars and other monosaccharides by the propanediol pathway mediated by various microbes, e.g., *Roseburia, Eubacterium* and *Blautia* species [38]. The gut partially metabolizes colon-derived propionate, and the liver uses the majority of it. Propionate is converted to propionyl-CoA by propionyl-CoA synthetase and then further metabolized to succinyl-CoA entering the TCA. Propionate is transported into the hepatocytes by organic anion transporters [41].

#### Acetate

Acetate may be formed from pyruvate after the release of CO_2_ (Fig. 2A). In addition, acetogenic bacteria utilize H_2_ to reduce CO_2_ to acetate. A wide range of gut microbes, e.g., *Bacteroides, Bifidobacterium, Clostridium, Ruminococcus* species, produce acetate [42, 43]. Acetate directly converts to acetyl-CoA that subsequently enters the mitochondrial TCA cycle to produce substrates for oxidative phosphorylation. The liver utilizes acetate to produce energy, cholesterol, long-chain fatty acids, glutamine and glutamate. From SCFA, acetate reaches the highest concentrations in the peripheral blood and thus contributes mostly to the systemic production of energy for the muscles, heart, kidney and adipose tissues [23, 44].

### Hydrogenotrophic products

#### Hydrogen sulfide

Gut bacteria can produce H_2_S from several substrates, particularly sulfate, sulfite, cysteine or taurine. The availability of colonic sulfate is determined mainly by the amount of substrates in the diet, e.g., inorganic sulfate, sulfur amino acids, and the absorption of substrates by the small intestine [45]. Sulfate reducing bacteria (SRB), predominantly *Desulfovibrio* species, generate H_2_S by the reduction of sulfate in the presence of electron donor to acetate or CO_2_ by incomplete and complete oxidation, respectively [46]. H_2_ is predominantly used as an electron donor, although other simple organic compounds (lactate, propionate, butyrate) may be utilized as well (Fig. 2B) [20]. The metabolism of sulfate is mediated by dissimilatory reduction coupled with the production of energy. In detail, sulfate is converted to adenosine phosphosulfate and further reduced to sulfite. Next, H_2_S is generated by the reduction of sulfite. The activation of sulfate is coupled with the consumption of ATP, while the reduction is coupled with the pumping of protons, thereby generating a proton gradient fueling the production of ATP. Dissimilatory sulfite reductase is a protein of very ancient origin. It is believed that sulfite metabolism occurred earlier than sulfate reduction and probably served to conserve energy in the first prokaryotes in the early earth [47].

Several bacterial species, e.g., *Escherichia, Salmonella, Enterobacter*, possess specific enzymes to produce H_2_S [48]. A phosphate-sulfite reductase maintains the redox balance by reducing sulfite to H_2_S and NADP^+^ or oxidizing H_2_S to sulfite and recovering NADPH [49]. Bacteria expressing cysteine desulfhydrase convert cysteine to H_2_S, pyruvate and ammonia [50]. *Bilophila wadsworthia* utilizes taurine by taurine:pyruvate aminotransferase to sulfoacetaldhyde and alanine, which is further metabolized by sulfoacetaldhyde sulfolyase to sulfite and acetate. Subsequent sulfite reduction by sulfite reductase produces H_2_S and CO_2_ [51]. Tissue enzymes also metabolize methionine to homocysteine, which is coupled with serine to form cystathionine by cystathionine β-synthase (CBS). Cystathionine γ-lyase (CSE) subsequently converts cystathionine to cysteine, which CBS or CSE may further transform to H_2_S. Half of the luminal H_2_S is produced by gut microbiota and half by colonic tissues [52]. Interestingly, the inhibition of the colonic tissue enzymes promotes the production of H_2_S by gut microbiota [52].

The proportion of dissociated HS^−^ to total sulfide is ~ 60% in the colon (pH ~ 7), the p*K*_a1_ = 6.8 and p*K*_a2_ > 12 [53]. The range of total sulfide in human feces is 0.2–3.4 mmol/l, averaging at 0.74 mmol/l [20]. H_2_S may exist in a free form or be bound to cysteine thiols as sulfane sulfur [54]. In addition, feces have large sulfide-binding activity and therefore, less than 8% of total sulfide (~ 60 µmol/l) is in the free form [20]. H_2_S is lipophilic and rapidly diffuses through lipid bilayers of cell membranes. Colonic mucosa effectively oxidizes H_2_S to thiosulfate [55]. A significant fraction of thiosulfate is recycled in the gut to H_2_S by the action of thiosulfate reductase expressed in several microbes, e.g., *Citrobacter, Proteus* and *Edwardsiella* [56, 57]. In addition, the oxidation products may be absorbed into the portal blood. We have shown that intracolonic injection of H_2_S increases thiosulfate in rats' portal blood. The fraction of thiosulfate that reached peripheral blood 30 min after the administration was ~ 80% lower than the fraction in the portal blood, suggesting a major role of the liver in the metabolism of gut-derived thiosulfate. In addition, the administration of antibiotics lowered the concentration of thiosulfate metabolites in the portal blood [58]. Shen et al*.* studied the concentrations of free sulfide and bound sulfane sulfur in the peripheral blood and tissues of germ-free mice. They found significantly lower levels of free H_2_S in the cecum, colon and peripheral blood plasma. The levels of sulfane sulfur were decreased in the blood plasma and adipose tissue of germ-free mice. Furthermore, lower activity of CSE and higher cysteine levels were found in gastrointestinal and extraintestinal tissues of germ-free mice [59].

**Fig. 3:**
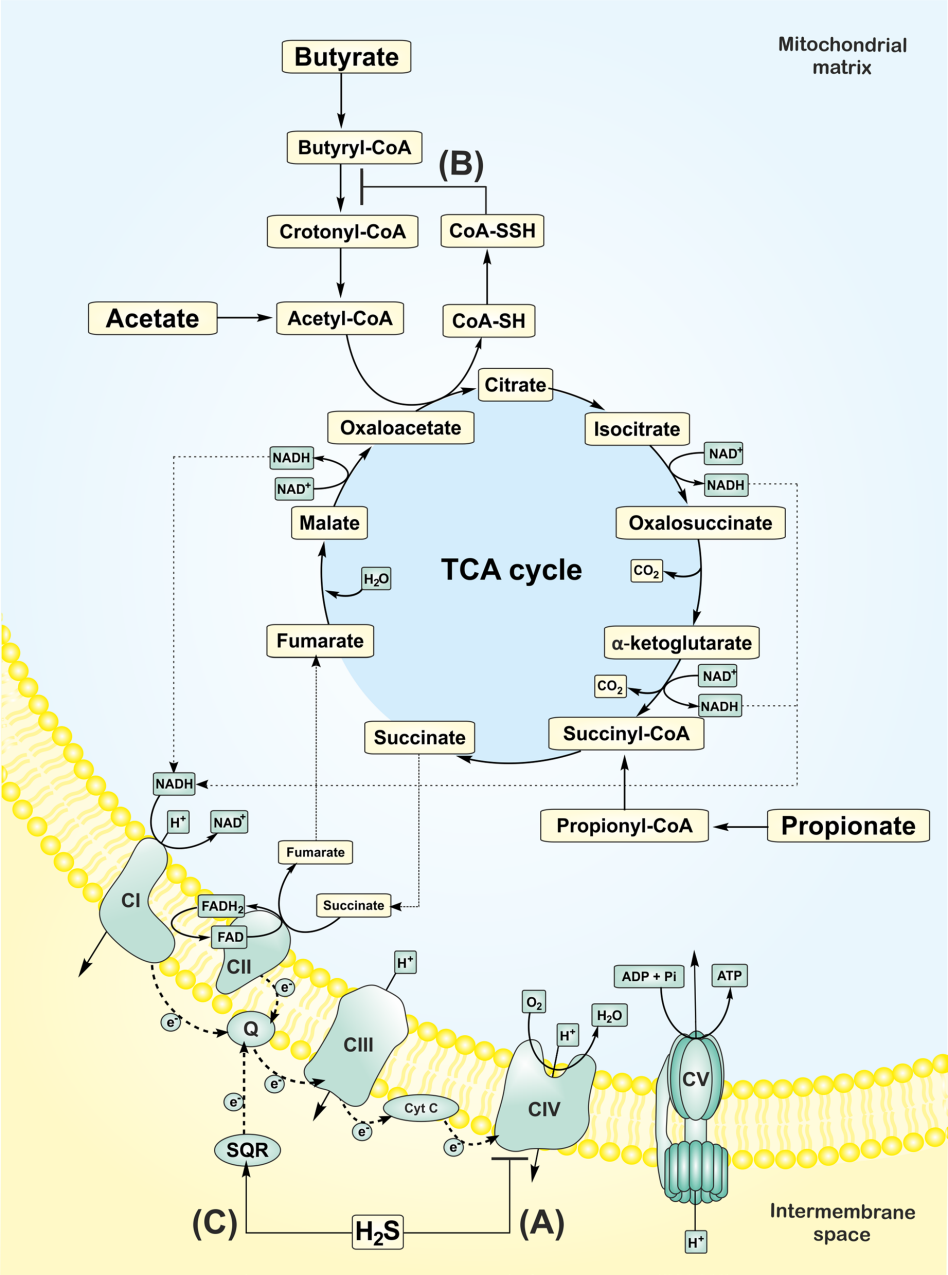
Effects of hydrogen sulfide (H_2_S) on mitochondrial bioenergetics. **A** At higher concentrations, H_2_S binds to complex IV (cytochrome *c* oxidase), thereby inhibiting mitochondrial electron transport and blocking ATP production. **B** A persulfide (-SSH) is formed on the cysteine residue (-SH) of coenzyme A in the presence of H_2_S, which blocks the oxidation and utilization of butyrate. **C** At low concentrations, mitochondria oxidize H_2_S by the action of sulfide SQR and transfer electrons through coenzyme Q and complexes III and IV to oxygen, thereby promoting ATP production at complex V (ATP synthase). Pathways have been simplified to highlight key end-products. CI-V; *complexes I-V*, e^−^; *electrons*, Q; *coenzyme Q*, cyt C; *cytochrome c*, CoA; *coenzyme A*, SQR; *sulfide quinone reductase*

The toxicological effects of H_2_S on mammals were studied over the centuries, with the first reports occurring in the eighteenth century. Toxic effects of H_2_S were described near swamps, volcanoes or industrial accidents [60]. H_2_S at ≥ 10 ppm causes eye and skin irritation and other neurological and cardiovascular disorders. The ability to sense the H_2_S odor disappears at ≥ 100 ppm due to the olfactory nerve's paralysis. H_2_S at ≥ 500 ppm causes respiratory arrest and death [61]. The toxic-effect mechanism is based on the reversible binding of H_2_S to the heme center of cytochrome *c* oxidase (complex IV). Thus, H_2_S competes with oxygen for the active site, resulting in the inhibition of the mitochondrial electron transport, loss of the transmembrane electrochemical gradient, and the reduction of ATP production (Fig. 3A) [62].

Additionally, H_2_S may block the β-oxidation of butyrate in the gut. Babidge et al. showed that H_2_S (1.5 mmol/l) strongly reduced the formation of CO_2_ from butyrate in colonocytes. In the presence of CoA and ATP, H_2_S decreased the formation of crotonyl-CoA and acetyl-CoA. It also increased the formation of butyryl-CoA, thus inhibiting the activity of butyryl-CoA dehydrogenase [63]. It should be stressed that the concentration used in this study was far over the physiological levels of free H_2_S reported in the rat colon (~ 120 µmol/l) [64]. Landry et al*.* showed that the oxidation of H_2_S by mitochondrial enzyme sulfide quinone reductase (SQR) results in the production of CoA persulfide (CoA-SSH), which inhibits the activity of butyryl-CoA dehydrogenase (Fig. 3B) [65].

Recent studies describe a bell-shaped effect of H_2_S on cellular functions. In detail, lower concentrations of H_2_S support cellular bioenergetics, whereas higher H_2_S concentrations block cellular respiration. First reports describing the utilization of H_2_S for ATP production in eukaryotic cells were performed in non-mammals. Recently, the promotion of bioenergetics was confirmed in various mammalian cells and isolated mitochondria [66–69]. The positive effect of H_2_S on functions of mitochondria isolated from rat liver was observed at 0.1–1 µmol/l H_2_S, whereas concentrations of H_2_S ≥ 3 µmol/l inhibited respiration and generation of ATP [66]. The concentrations of H_2_S promoting mitochondrial function in colonocytes were much higher (20–40 µmol/l) in comparison to other cell types [14]. The inhibition of the colonocytes' oxygen consumption occurred at a concentration ≥ 65 µmol/l [14]. In addition, exogenous H_2_S promoted the production of colonic mucus and the formation of microbial biofilms [70]. These reports suggest that the colon is adapted to the H_2_S-rich environment and utilizes H_2_S to protect the integrity of the gut-blood barrier. Since the average concentration of free H_2_S in human colon lumen was estimated to be 60 µmol/l, most individuals may employ H_2_S to produce energy [20]. However, in individuals with a concentration of free H_2_S over 65 µmol/l, inhibition of colonocytes respiration may occur. It should be noted that several common detection methods used in biological samples overestimate physiological levels of H_2_S due to the additional liberation of H_2_S from the bound stores [71]. Interestingly, mitochondrial enzymes can also oxidize H_2_S, a function that can be coupled with ATP generation [72]. One of the evolution theories posits that eukaryotes evolved from an endosymbiotic ancestral mitochondrion living in a prokaryotic host. The symbiotic relationship was based on the oxidation of H_2_S by the ancestral mitochondrion coupled with the reduction of oxidized sulfur forms to H_2_S by the host prokaryote [73]. This hypothesis is also supported by the fact that mitochondria share common features with a purple sulfur bacterium Rhodobacter [74]. In modern eukaryotic cells, mitochondria utilize electrons from H_2_S through SQR to coenzyme Q [66]. Electrons are subsequently driven through complex III and IV to oxygen, resulting in ATP production at complex V (Fig. 3C). The oxidation of H_2_S by SQR results in the formation of sulfane sulfur at the SQR cysteine group (SQR-SSH). The sulfane sulfur may be further transferred to glutathione to form glutathione persulfide or to sulfite to form thiosulfate. Persulfide dioxygenase (ETHE1) and thiosulfate sulfurtranferase subsequently oxidize the persulfides to reduce coenzyme Q and regenerate sulfite, thereby consuming oxygen. Finally, sulfite oxidase converts sulfite to sulfate [75]. Bound sulfane sulfur forms have recently attracted significant attention as an H_2_S pool and signaling molecules. Akaike’s group identified an endogenous source of cysteine persulfide (Cys-SSH) in the mitochondria, the mammalian cysteinyl-tRNA synthetase 2 (CARS2). They showed that mitochondrial respiration is lower in CARS2 KO cells. They provided further evidence of the reduction of CARS2 derived Cys-SSH by accepting an electron from the mitochondrial electron transport chain to produce H_2_S [76].

#### Nitric oxide-species

Nitric oxide species (NOx) are produced and utilized by commensal bacteria via various pathways. Firstly, oral bacteria can convert nitrate (NO_3_^−^) to nitrite (NO_2_^−^) by nitrate reductases [77]. Subsequently, under the stomach's acidic environment, the chemical reduction of nitrite generates NO. Secondly, gut bacteria can reduce nitrate either by respiratory denitrification or by dissimilatory and assimilatory nitrate reduction (Fig. 2B). In the denitrification process, the reduction of nitrate by membrane-bound nitrate reductases to nitrogen oxides (NO and N_2_O) and N_2_ gas leads to the translocation of protons and generation of ATP [77]. The dissimilatory nitrate reduction to ammonium (DNRA) by nitrate reductases located in the periplasm is not directly associated with energy conservation [78]. The coupling of DNRA with the oxidation of substrates, such as formate or lactate, generates ATP [79]. Assimilatory nitrate reduction by cytosolic nitrate reductases to ammonium is coupled with incorporating the derived ammonium to glutamine or glutamate. The reduction of nitrate may be either non-enzymatic or catalyzed by nitrite/nitrate reductases [80, 81]. Several microbial species express bacterial nitric oxide synthase (bNOS) [82–84]. Compared to eukaryotic isoforms, bNOS is smaller and lacks a reductase domain, which supplies electrons for NO synthesis. Therefore, other bacterial reductases must couple with bNOS to provide electrons for NO synthesis [84].

Analysis of human fecal samples determined the concentration of 5–19 µmol/kg and 0–14 µmol/kg for nitrite and nitrate, respectively [85]. The colon tissue of rats contained 1–1.5 µmol/l of nitrite and 4–32 µmol/l of nitrate [86]. Whitter et al*.* studied the distribution of labeled nitrate and nitrite in rats. The labeled ^13^N was found in the liver, kidney, cecum, large intestine, and blood, however not in rats' feces after gavage of labeled nitrate and nitrite. The absorption from the stomach was faster for nitrate (70%) than for nitrite (39%) [87]. The fraction of absorbed nitrite from the colonic lumen either entered the circulation or was oxidized in the colonocytes and partly reentered the colonic lumen as nitrate [88]. The plasmatic levels of nitrate in germ-free mice were strongly reduced [89]. Surprisingly, nitrite levels in plasma were increased in germ-free mice after nitrate supplementation, probably due to the activation of nitrate reduction in the liver as a compensatory response to the absence of microbiota [90]. Also, the distribution of labeled nitrate administered by gavage was altered in germ-free rats, with lower levels of ^13^N found in the gut and higher levels in the stomach and bladder [87]. The absorption of labeled nitrate administered into the lower intestines of germ-free rats was faster than the absorption in conventional rats. In reverse, the absorption of nitrate from circulation to the intestinal lumen was lower in germ-free rats than in control animals [87].

Similar to H_2_S, the inhibitory effect of NO on mitochondrial respiration has been known for centuries. NO interacts at physiological levels (1–200 nmol/l) with the oxygen-binding site of the cytochrome *c* oxidase, thereby rapidly but reversibly inhibiting the enzyme's activity. The binding of NO may occur at the reduced heme center of the cytochrome *c* oxidase, thus competing with oxygen binding or at the oxidized copper center by nitrite. The inhibition of mitochondrial functions at higher concentrations of NO (≥ 1 µmol/l) and by reactive nitrogen species, particularly peroxynitrite, is slow and non-selective but irreversible due to the modification of proteins. On the other hand, NO/cGMP signaling stimulates mitochondrial biogenesis through the activation of PGC-1α [91]. Nitrite (5 mmol/l) promotes the oxidation of butyrate in rat colonic cells [92]. It should be stressed that the concentration used in this study was far over the physiological levels of nitrite reported in the rat colon (~ 1 µmol/l). Larsen et al*.* showed that supplementation with nitrate decreased whole-body oxygen consumption during exercise. This finding strongly correlated with the increased efficiency of oxidative phosphorylation in mitochondria isolated from human skeletal muscle. The basal respiration, proton leak and expression of ATP/ADP translocase were decreased in human skeletal muscle after nitrate supplementation, suggesting that the increase in mitochondrial efficiency could be mediated by reduced leakage of protons across the inner mitochondrial membrane.

Interestingly, nitrate supplementation increased the plasmatic levels of nitrite. However, the possible involvement of commensal microbes in the reduction of nitrate to nitrite was not examined. In contrast to nitrate, nitrite did not affect basal respiration or oxidative phosphorylation and decreased oxygen affinity to skeletal muscle mitochondria at pH 6.7, but not at pH 7.2. Since hypoxia and low pH facilitate the conversion of nitrite to NO, it is assumed that NO-dependent mitochondrial inhibition mediates the effect [93].

#### Methane and methylamines

Methane derivates are produced in the human gut by methanogenic, anaerobic archaea. Methanogens express methyl-coenzyme M reductase, which catalyzes the formation of methane. The majority of methanogens are hydrogenotrophic and utilize H_2_ to reduce CO_2_ to methane (Fig. 2B). Alternatively, methylotrophs convert methylated compounds, e.g., methanol, methylamines and methyl-sulfides to methane by substrate-specific methyltransferases [94]. The human gut's dominant methanogen is *Methaninobrevibacter smithii*, which preferentially colonizes the distal portion of the colon [95]. Methane is excreted in the breath and flatus or metabolized in the liver after absorption through the gut-blood barrier. Interestingly, despite changes in diet over a period of 35 years, human methanogens' activity remained stable [96]. The determination of methane in human breath divides the subject into the group of methane producers (~ 35%), with the mean level of methane ~ 15 ppm, and methane nonproducers, with concentrations of < 1 ppm [96]. The methane production status is similar between family members. In addition, methane excretion was not detected in germ-free rats nor infants up to 6 months of age, nor was it significantly altered by administering antibiotics [97].

In addition, dietary carnitine and choline degradation results in the production of trimethylamine (TMA) in the gut. Various members of human gut microbiota mediate the conversion of substrates to TMA, e.g., *Anaerococcus, Clostridium, Desulfitobacterium, Escherichia, Proteus, Providencia, Edwardsiella, Yokenella and Citrobacter* species [98, 99]. TMA may be further utilized by methylotrophs from Methanomassiliicoccales genera in the gut or pass through the gut-blood barrier to the portal vein and reach the liver [100, 101]. Flavin monooxygenase 3 (FMO-3) oxidizes TMA to trimethylamine N-oxide (TMAO) in the liver, which the circulation transports for excretion in the urine, sweat and breath. The concentration of TMA in the colon and TMAO in the plasma of healthy humans ranges between 1–200 μmol/l and 3–4 μmol/l, respectively [102, 103]. The administration of antibiotics significantly reduced plasma TMAO. Moreover, TMA and TMAO were below the detection level in germ-free mice [104].

Alcohol intake reduced mitochondrial respiration and increased whole-body methane emission in humans and rats [105]. However, incubation of liver mitochondria with methane did not alter mitochondrial respiration [13]. A protective effect of methane was observed in an experimental model of ischemia–reperfusion injury of the liver. Methane inhalation reduced cytochrome *c* oxidase release and improved basal respiration and maximal respiratory capacity in liver mitochondria during ischemia–reperfusion [13]. Makrecka-Kuka et al*.* studied the effect of TMAO on the respiration of cardiac mitochondria. They showed that acute exposure to TMAO decreased oxidative phosphorylation in cardiac fibers. The activity of pyruvate dehydrogenase was also reduced. Additionally, treatment of mice with TMAO for 8 weeks impaired cardiac mitochondrial energy metabolism [106]

## Co-colonization and competition between gut microbes

SRB co-colonize with other species, which produce substrates for sulfate reduction. For instance, SRB are abundant in the presence of *Bacteroides* that generate sulfate from sulfomucin and mucopolysaccharides by sulfatases (*B. thetaiotaomicron, B. fragilis*) [107]. The levels of *D. piger* positively correlate with the levels of an H_2_ producer *Collinsella aerofaciens*. This co-colonization is beneficial for both strains because the removal of H_2_ by *D. piger* increases the efficiency of dietary fermentation by *C. aerofaciens* [108].

A negative correlation was observed between SRB and hydrogenotrophic methanogens, which compete for the common substrate H_2_ [109]. Co-incubation of human SRB with methanogens reduced the production of H_2_S in the feces; however, it did not alter the production of methane [110]. Supplementing the diet with sulfate increased both sulfate reduction in the subjects' feces and the number of SRB, which were undetectable during control diets. The number of methanogenic bacteria and methane expiration decreased after supplementing a sulfate diet [111]. Another mechanism is the conversion of choline to TMA by *Desulfovibrio desulfuricans*, which promotes the metabolism of methylotrophic methanogens over hydrogenotrophic methanogens [112].

Several studies suggest that SRB may utilize nitrate and nitrite as electron acceptors. The data implies that SRBs generate ATP and, at the same time, compete with denitrifying and nitrate fermenting bacteria for these substrates [113]. On the other hand, the presence of nitrate and nitrite suppresses the reduction of sulfate by SRB [113]. The dissimilatory reduction of nitrate (DNRA) and nitrite to ammonium are not generally attributed to SRB. Only a few strains of *Desulfovibrio* species possess this ability. It was reported that *Desulfovibrio desulfuricans* express nitrite reductase constitutively, and this expression of nitrate reductase is inducible by nitrate or nitrite and suppressed by sulfate [113]. The reduction of nitrate to ammonia by *D. desulfuricans* and *Sulfurospirillum deleyianum* was coupled with the oxidation of H_2_S to sulfate or elemental sulfur, respectively [114, 115]. Interestingly, *M. smithii* may utilize ammonium as a primary nitrogen source, competing with *B. thetaiotaomicron* for this substrate [116].

## Gut dysbiosis, energy accumulation and related diseases

Excessive energy accumulation in the body is associated with the development of cardiometabolic disorders, such as obesity, diabetes and metabolic syndrome [117]. Gut microbes play an essential role in the regulation of the host's energy homeostasis. Alterations in the composition of gut microbes are associated with disturbed energy balance resulting in the development of various diseases [118]. These pathologies or phenotypes may be related to an overall reduction in microbial diversity and an imbalance of microbial composition. The result of which may be either an overproduction of some metabolites and/or suppression of others. Below, we review evidence on the involvement of gut microbiota metabolites in the pathogenesis of metabolic diseases and their cardiovascular complications.

### Obesity and diabetes

**Fig. 4:**
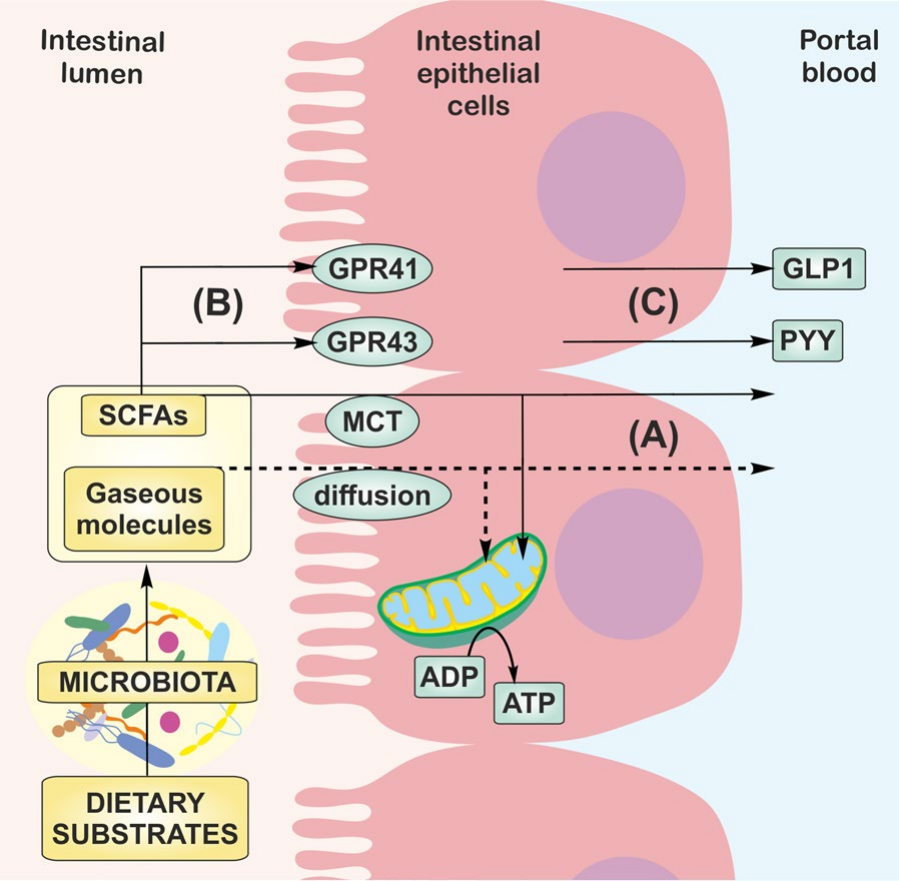
Transport and signaling of gut microbiota products. **A** SCFAs are transported by MCT, while, gaseous molecules diffuse to the colonic cells and are oxidized in the mitochondria or in the unmetabolized form enter portal vein. **B** SCFAs stimulate GPR41 and GPR43 and **C** the secretion of gut hormones, GLP-1 and PYY, which regulate energy homeostasis in the body. MCT; *monocarboxylate transporters*, GPR; *G-protein coupled receptor*, GLP-1; *glucagon-like peptide-1*, PYY; *peptide YY*

Mitochondrial dysfunction is a major factor leading to the accumulation of lipids and decreased sensitivity to insulin [117]. Based on metabolomic studies, Samczuk et al. suggested a relationship between mitochondria, gut microbiota metabolites and clinical outcome in obese patients with diabetes undergoing bariatric surgery. In this group, rapid improvement in blood glucose control was associated with changes in mitochondrial citrate cycle pathway, ketone bodies synthesis and degradation, butyrate and propionate metabolism whereas patients with slower improvement of glucose control showed alternations in nitrogen metabolism, branched-chain fatty acids (BCFAs) synthesis and degradation [119]. In animal studies, administration of butyrate and nitrate improved mitochondrial functions in liver, skeletal muscle and adipose tissue of insulin-resistant obese mice [120–124]. In addition, accumulating evidence suggests that gut metabolites affect lipid, glucose and cholesterol metabolism in peripheral tissues [18, 22, 125–128]. Germ-free mice colonized with microbiota from obese donors show higher weight gain and total body fat than germ-free mice colonized with microbiota from lean mice [129]. The oxidation of SCFAs is associated with the inhibition of lipolysis and de novo synthesis of fatty acids resulting in reduced plasmatic concentrations of free fatty acids in the plasma [130]. Besides the direct effect on mitochondrial respiration, SCFAs regulate energy homeostasis via G-protein coupled receptors 41 (GPR41) and 43 (GPR43), also known as free fatty acid receptor 3 (FFAR3) and 2 (FFAR2). In addition, SCFAs stimulate the secretion of gut hormones, peptide YY (PYY) and glucagon-like peptide-1 (GLP-1), which modulate the response of brain centers responsible for food intake (Fig. 4). Lower levels of acetate were found in the liver and brain of mice on a high-fat diet [131]. The administration of acetate reduced the accumulation of lipids in the adipose tissue and liver and improved glucose tolerance in obese animals [132, 133]. Acetate is taken up by the brain and directly modulates appetite in hypothalamic centers [134].

On the other hand, propionate stimulates the accumulation of PYY and GLP-1 in the portal vein and the expression of GPR43; thus, promoting intestinal gluconeogenesis and reducing food intake [135]. Obesity and diabetes are also associated with lower bioavailability of NO [22]. Moreover, the administration of nitrate or nitrite reduces the accumulation of fat in adipose tissues and improves insulin signaling in mouse models of diabetes and metabolic syndrome [136, 137].

Various studies suggest that the levels of circulating metabolites positively correlate with the development of metabolic disorders [22, 138–140]. Higher levels of BCFAs were detected in obese individuals and subjects on a high-fat diet. Similarly, a more significant release of H_2_S from adipose tissue was detected in animals on a high-fat diet [141]. Administering H_2_S stimulated lipolysis, promoted energy storage in adipocytes via the peroxisome proliferator-activated receptor γ (PPARγ) pathway, and marginally increased fat accumulation in visceral tissues of animals on a high-fat diet [142]. Several reports also describe the inhibitory effect of H_2_S on glucose-stimulated insulin secretion as a factor contributing to the development of diabetes. This effect is mediated via the opening of ATP-sensitive potassium channels (K_ATP_) [22]. TMAO attracted attention in recent years as a marker of cardiovascular health. A systemic review showed that the risk of major cardiovascular events and all-cause mortality is higher in patients with high TMAO plasma levels than patients with low TMAO levels [143]. One of the mechanisms responsible for higher cardiovascular risk involves the suppression of cholesterol metabolism by TMAO. In detail, TMAO inhibits the reverse transport of cholesterol and the synthesis of bile acids in the liver [144]. It should be pointed out that the changes in the plasmatic levels of metabolites were not associated with changes in the gut microbiota composition and could have resulted from altered tissue metabolism.

### Hypertension

High blood pressure is one of the hallmarks of metabolic syndrome. It increases the risk of developing diabetes and obesity. Recent studies suggest that gut microbes are involved in the regulation of blood pressure. Hypotension and reduced vascular contractility were observed in germ-free rats [145]. In addition, angiotensin II-induced increase in blood pressure was lower in germ-free mice than conventionally raised mice [146], suggesting that gut microbiota promotes the development of hypertension. Several other gut-derived products were shown to exert antihypertensive actions. Acute or chronic administration of SCFAs, acetate, propionate, butyrate and valerate, lowered blood pressure in mice and rats [147–150]. The activation of GPR41 was proposed as the underlying mechanism of the SCFAs-mediated hypotensive effects [148, 151].

NO and H_2_S mediated vasodilation and blood pressure-lowering effects are known over decades. However, the role of microbiota-derived H_2_S and NO-species in the regulation of blood pressure remains unclear. We have shown that the administration of H_2_S into the colon lowered arterial blood pressure several times longer than parenteral administration. Furthermore, the hypotensive effect of colonic H_2_S was more pronounced in hypertensive animals than normotensive controls [58]. Daliri et al*.* reported that consumption of soy protein decreased blood pressure, possibly by increasing the colonization of the gut by H_2_S-producing bacteria in hypertensive rats [152]. Similarly, dietary nitrate reduced blood pressure in healthy volunteers and hypertensive patients [18]. In contrast, hypertensive rats showed a greater permeability of the colon to TMA, and TMA administration increased blood pressure in rats [153, 154]. Although plasma TMAO levels correlate with a higher risk of hypertension and preeclampsia, growing evidence suggests that gut bacteria-produced TMA exerts toxic effects; that is, TMAO precursor is the culprit rather than TMAO itself [103, 155, 156].

## Conclusion

During dietary fermentation, gut microbes metabolize dietary substrates to gain energy most efficiently, thereby maintaining redox balance. This process generates a wide variety of products. The most abundant are SCFAs, which represent the primary fuel for colonic epithelial cells. SCFAs also play a crucial role in the regulation of energy homeostasis in peripheral tissues.

H_2_S, together with other gut-derived gases and their precursors, emerged as signaling molecules capable of influencing cellular bioenergetics. These compounds may diffuse into the cell and directly affect energy production. They may also interact with the production and metabolism of other gut metabolites, particularly SCFAs. Despite the accumulating evidence demonstrating the involvement of gut-derived metabolites in cellular energy production, more research is needed to elucidate the role of these compounds in regulating host energy metabolism. More specifically, changes in the composition of gut microbes and their products should be studied in relation to different lifestyle disorders to establish a link between gut dysbiosis and the development of metabolic diseases. In order to create therapeutics based on gut microbial interventions, detailed mechanisms by which these organisms contribute to the regulation of energy metabolism need to be described.

## Data Availability

Not applicable.

## Abbreviations

BCFAs: Branched-chain fatty acids
bNOS: Bacterial nitric oxide synthase
CARS2: Cysteinyl-tRNA synthetase 2
CBS: Cystathionine β-synthase
CoA: Coenzyme A
CSE: Cystathionine γ-lyase
cyt C: Cytochrome c
DNRA: Dissimilatory nitrate reduction to ammonium
ETHE1: Persulfide dioxygenase
FFAR: Free fatty acid receptor
FMO-3: Flavin monooxygenase 3
GLP-1: Glucagon-like peptide-1
GPR: G-protein coupled receptor
H_2_S: Hydrogen sulfide
K_ATP_: ATP-sensitive potassium channels
MCT: Monocarboxylate transporters
NO: Nitric oxide
NOx: Nitric oxide species
PPARγ: Peroxisome proliferator-activated receptor γ
PYY: Peptide YY
SQR: Sulfide quinone reductase
SRB: Sulfate-reducing bacteria
TMA: Trimethylamine
TMAO: Trimethylamine N-oxide
